# The Impact of Cosmetic Surgery on Women’s Marital Satisfaction and Self-Concept

**DOI:** 10.29252/wjps.7.3.337

**Published:** 2018-09

**Authors:** Nazanin Rita Davai, Abdoljalil Kalantar-Hormozi, Kamran Ganji, Ali Abbaszadeh-Kasbi

**Affiliations:** 1 Department of Clinical Psychology, Private Practice, Tehran, Iran;; 2 Department of Plastic and Craniofacial Surgery, Medical College, Shahid Beheshti University of Medical Science, 15 Khordad Hospital, Tehran, Iran;; 3 Department of Psychology, Malayer Branch, Islamic Azad University, Malayer, Iran;; 4 School of Medicine, Tehran University of Medical Sciences, Tehran, Iran

**Keywords:** Marital satisfaction, Self-concept, Women, Cosmetic surgery

## Abstract

**BACKGROUND:**

Today, cosmetic surgery is one of the most common procedures worldwide, and its use is increasing. This study aimed to identify the impact of cosmetic surgery on married women’s marital satisfaction and self-concept.

**METHODS:**

From January 1^st^, 2015 to June 6^th^, 2015, 44 married women operated in a plastic surgery clinic or seeking for a cosmetic surgery and 55 non-applicants as general population were enrolled. ENRICH marital satisfaction questionnaire and Rogers’ Self-concept Inventory were used to compare groups.

**RESULTS:**

Operated applicants revealed a significant higher level of satisfaction in comparison with other groups regarding personality, conflict and leisure. Compared to control group, the surgical group had significantly higher satisfaction in marital and familial relationship, friendship, and financial management.

**CONCLUSION:**

It can be drawn that expectation for postoperative positive outcome probably is an important factor affecting high level of marital satisfaction in cosmetic surgery applicants. Furthermore, the self-concept is significant predictor of applying for cosmetic surgery.

## INTRODUCTION

Today, cosmetic surgery is one of the most common procedures worldwide, and its use is increasing.^1^ The frequency of plastic surgery in 2005 was 10.2 million in United States and increased to 11.7 million in 2007.^2^^,^^3^ In Iran, there has been about 70 percent increase in demand for cosmetic surgery since 1990s, and this rate has an increasing trend.^4^ Marital satisfaction has been defined as objective feeling of happiness, satisfaction and joy experienced by the couples, when considering all aspects of marriage. Marital satisfaction is an attitudinal variable; therefore, it is regarded as couple’s personal attributes. One of the variables affecting the association between cosmetic surgery and marital satisfaction is physical attractiveness.^4^

It was shown that factors predicting the likelihood of demand for unnecessary (non-indicated) cosmetic surgery and showed that the lower levels of physical attractiveness (self-evaluation) predict the high demand for cosmetic surgery; therefore, it seems that one of the factors that the married cosmetic surgery applicants follow is enhancing physical attractiveness (this probably justifies the demand for women cosmetic surgeries including breast surgery and the like) and promoting marital satisfaction.^5^

Those who feel they are not physically attractive are more likely to undergo cosmetic surgeries. This idea supports the idea that failure to achieve the social ideals of attractiveness results in higher levels of physical dissatisfaction and finally leads to cosmetic surgeries. It ought to be noted that higher levels of physical satisfaction will not be achieved by these people after the surgery, if these demands are due to these individuals’ low self-estimate. This reveals the critical role of another variable mediating the impact of cosmetic surgery on expected consequence (i.e. self-concept).

Self-concept is a dynamic system of beliefs, values, sentiments, talents and capabilities. Self-concept refers to one’s self-assessment. This is an individual’s subjective assessment of its own characteristics which may be positive or negative. Positive self-concept indicates that the person accept himself as a person having strengths and weaknesses, leading to his enhance self-confidence in social relations. Negative self-concept reflects the feelings of worthlessness, incompetence and disability.^6^

Self-concept is an overall perception of what we are.^7^ Self-concept is defined as one’s image of himself including actual experiences and the interpretation of these experiences. In general, self-concept is a multi-dimensional and multi-level concept. Body image and self-esteem are studied as the most vital aspects of self-concept.^8^ A large number of studies have dealt with the relationship between body image and self-concept and psychological variables. Some studies revealed the relationship between body image and societal perfectionism and internalization of social messages, the need to hide perceived flaws from others, perfectionism, and psychological damages.^9^^-^^12^

Several studies indicated the relationship between self-concept and social functioning, individual goal orientation and the demand for cosmetic surgery.^13^^-^^15^ Here, the impact of cosmetic surgery on marital satisfaction and self-concept as probable factors affecting the demand for cosmetic surgery has been assessed. Investigating this issue is necessary to determine clinical features of this specific population in order to prevent unnecessary surgery leading to postoperative adverse outcomes.

## MATERIALS AND METHODS

The study population is consisted of married women who had or requesting for cosmetic surgery from January 1^st^, 2015 to June 6^th^, 2015. Inclusion criteria were consent and cooperation. The surgery date was specified by plastic surgeon after passing medical procedures and having no facial cosmetic surgery and medical and urgent reasons to perform cosmetic surgery for applicants. Exclusion criteria were having no diagnosable mental disorder as well as not having cosmetic surgery on one body organ for the second time. The control group was non-applicants who were selected from the volunteers’ relatives. The research sample was consisted of 44 individuals who had facial cosmetic operation, 51 individuals requesting for cosmetic surgery and 55 were control as non-applicants from general population. 

Instruments that were used included (i) ENRICH Marital Satisfaction Questionnaire. The scale was developed before to assess potentially problematic areas and areas of strengths and in marital relations. ENRICH Questionnaire contains 12 subscales as follows: Idealistic distortion, marital satisfaction, personality issues, communication, conflict resolution, financial management, leisure activities, intercourses, children, parenting, family and friends, egalitarianism roles and religious orientation. This questionnaire is composed of strong psychometric properties.^15^ Olson *et al.*^16^ estimated the internal consistency of this questionnaire from 0.73 to 0.90. 

(ii) Rogers’ Self-concept inventory developed in 1951 was a determinant of individuals’ positive and negative self-concept. The time required to answer this test was approximately 20 minutes. It is an objective test and is consisted of a 7-score level assigned between two features and one is supposed to select one of the numbers between the two features. This test has A and B forms. During the presentation of Form A, the participants were asked to mark a number based on the given scale (ranging from one to seven based on closeness to the supposed feature). Then, the participants were asked to specify their ideal personal traits in the Form B. The calculated scores between 0-7 and 7 and above indicate normal and negative/weak self-concept, respectively. In other words, high scores obtained for this scale show a mismatch between the actual self and the ideal self. 

Pasha *et al.*^17^ in their study reported the reliability of this scale using split-half method and Cronbach’s alpha can be 0.79 for the Form A and 0.55 for the Form B, generally indicating acceptable values. Raghibi *et al.*^18^ showed that the Cronbach’s alpha values were estimated between 0.69 and 0.60 for the Form A and Form B, respectively. In order to analyze the data, descriptive statistics as well as one-way analysis of variance (ANOVA) and multivariate analysis of variance (MANOVA) were run with the SPSS software (version 21, Chicago, IL, USA).

## RESULTS

Forty (85%) women underwent cosmetic surgery, 32 (80%) of women looked for cosmetic surgery, and 39 (86%) were control group with academic education. Moreover, the data revealed that the age range in 16 (34%) women undergoing cosmetic surgery, 19 (46.3%) women seeking cosmetic surgery and 21 (46.7%) of women of control group were between 21 and 40 years. Also, 26 (55.3%) women who had cosmetic surgery, 18 (43.9%) women seeking cosmetic surgery and 21 (46.7%) women of control group were 41 years of age and older. The mean age of women in operated group, applicants seeking for surgery, and control group were 42.26±5.77, 39.58±6.63, and 40.91±5.6 yeas, respectively.

According to the variance analysis results shown in Table 1, significant differences were observed among the subjects in the three groups of undergoing surgery, applicant for operation and control in terms of all the marital satisfaction components except for the components of sexual relations, marriage, children, and religious orientation. Furthermore, the three groups were significantly different in terms of the total marital satisfaction score (*p*<0.001). 

**Table 1:** Results of ANOVA on mean marital satisfaction components

**Variable**	**Source**	**df**	**Mean Square**	**F**	**P value**
Personality Issues	Between Groups	2	4.718	6.589	<0.001*
	Within Groups	147	0.716		
	Total	149			
Communication	Between Groups	2	2.655	3.664	0.02*
	Within Groups	147	0.724		
	Total	149			
Conflict Resolution	Between Groups	2	4.610	8.095	<0.001*
	Within Groups	147	0.570		
	Total	149			
Financial Management	Between Groups	2	2.419	3.778	0.02*
	Within Groups	147	0.640		
	Total	149			
Leisure Activities	Between Groups	2	3.862	5.667	<0.001*
	Within Groups	147	0.682		
	Total	149			
Sexual Relationship	Between Groups	2	2.096	1.769	0.17
	Within Groups	147	1.184		
	Total	149			
Children and Parenting	Between Groups	2	0.212	0.483	0.61
	Within Groups	147	0.438		
	Total	149			
Family and Friends	Between Groups	2	2.094	4.449	0.01*
	Within Groups	147	0.471		
	Total	149			
Religious Orientation	Between Groups	2	0.904	1.604	0.20
	Within Groups	147	0.563		
	Total	149			
Overall marital satisfaction	Between Groups	2	2.146	6.424	<0.001*
	Within Groups	147	0.334		
	Total	149			

As shown in Table 2, the surgery group and the applicant group for operation were significantly different from each other in terms of the adjusted marital satisfaction mean scores; this means that the participants in the applicant group reported higher marital satisfaction. Moreover, the adjusted mean scores of the marital satisfaction components were significantly different in the applicant group, as compared with both the surgery and control group, while, the applicant subjects reported higher satisfaction in majority of the components. 

**Table 2:** Results of comparison of pairs of adjusted means of the marital satisfaction components at the group level using Tukey’s test between the group seeking and undergone for cosmetic surgery and the non-applicant control group

**Components of marital satisfaction**	**Groups**	**P value**
Personalit issues	Requesting vs. undergone for cosmetic surgery	0.01*
	Non-applicants vs. undergone for cosmetic surgery	0.89
	Non-applicants vs. requesting for cosmetic surgery	<0.001*
Communication	Requesting vs. undergone for cosmetic surgery	0.11
	Non-applicants vs. undergone for cosmetic surgery	0.90
	Non-applicants vs. requesting for cosmetic surgery	0.03*
Conflict resolution	Requesting for cosmetic surgery vs. who had surgery	<0.001*
	Non-applicants vs. who had surgery	0.85
	Non-applicants vs. requesting for cosmetic surgery	<0.001*
Financial management	Requesting vs. undergone for cosmetic surgery	0.02*
	Non-applicants vs. undergone for cosmetic surgery	0.53
	Non-applicants vs. requesting for cosmetic surgery	0.18
Leisure activities	Requesting vs. undergone for cosmetic surgery	0.03*
	Non-applicants vs. who had surgery	0.90
	Non-applicants vs. requesting for cosmetic surgery	<0.001*
Sexual relationship	Requesting for cosmetic surgery vs. who had surgery	0.81
	Non-applicants vs. undergone for cosmetic surgery	0.48
	Non-applicants vs. requesting for cosmetic surgery	0.15
Children and parenting	Requesting for cosmetic surgery vs. who had surgery	0.60
	Non-applicants vs. undergone for cosmetic surgery	0.76
	Non-applicants vs. requesting for cosmetic surgery	0.95
Family and friends	Requesting vs. undergone for cosmetic surgery	0.01*
	Non-applicants vs. who had surgery	0.48
	Non-applicants vs. requesting for cosmetic surgery	0.13
Religious orientation	Requesting vs. undergone for cosmetic surgery	0.17
	Non-applicants vs. undergone for cosmetic surgery	0.61
	Non-applicants vs. requesting for cosmetic surgery	0.63
Overall marital satisfaction	Requesting vs. undergone for cosmetic surgery	<0.001*
	Non-applicants vs. undergone for cosmetic surgery	0.98
	Non-applicants vs. requesting for cosmetic surgery	<0.001*

Accordingly, the applicant group reported higher satisfaction than the surgery and control group in terms of the personality issues component, higher satisfaction than the control group regarding communication component, higher satisfaction than the surgery and control group regarding conflict resolution and leisure activities components, and higher satisfaction than the surgery group in terms of the financial management and family and friends’ components. Expectation of positive outcomes in the applicant group after cosmetic surgery had a positive impact, although can be transient, on the marital satisfaction components. 

Table 3 demonstrates that significant differences were observed among the subjects in the three groups for ideal self-concept and overall self-concept score; however, these groups were observed to be similar regarding the actual self-concept score. Therefore, various levels of self-concept can be concluded in people undergoing cosmetic surgery, surgery applicants and ordinary people. As revealed in Table 4, no significant difference was observed in the adjusted real self-concept mean scores between the three groups of operated, surgery applicant and the control. 

**Table 3:** Results of ANOVA on the mean score of actual self-concept, ideal self-concept and self-concept

**Variable**	**Source**	**df**	**Mean Square**	**F**	**P value**
Real self-concept	Between Groups	2	74.228	0.713	0.49
	Within Groups	147	104.078		
	Total	149			
Ideal self-concept	Between Groups	2	453.152	4.089	0.01*
	Within Groups	147	110.811		
	Total	149			
Self-concept	Between Groups	2	597.420	9.147	<0.005*
	Within Groups	147	65.310		
	Total	149			

* Indicates that value is statistically significant.

**Table 4:** Results of comparison of pairs of adjusted means of actual self-concept, ideal self-concept and self-concept at the group level using Tukey’s test

**Components of marital satisfaction**	**Groups**	**P value**
Real Self-concept	Requesting for cosmetic surgery vs. undergone for cosmetic surgery	0.58
	Non-applicants vs. undergone for cosmetic surgery	1.0
	Non-applicants vs. requesting for cosmetic surgery	0.53
Ideal Self-concept	Requesting for cosmetic surgery vs. undergone for cosmetic surgery	0.50
	Non-applicants vs. undergone for cosmetic surgery	0.01*
	Non-applicants vs. requesting for cosmetic surgery	0.19
Self-concept	Requesting for cosmetic surgery vs. undergone for cosmetic surgery	0.97
	Non-applicants vs. undergone for cosmetic surgery	<0.001*
	Non-applicants vs. applying for cosmetic surgery	<0.001*

* Indicates that value is statistically significant

Regarding ideal self-concept, however, a significant difference was noted in the surgery group when compared to the control group. Moreover, regarding the final self-concept score, resulting from the difference between real and ideal self-concept, the control group demonstrated higher positive self-concept compared to other groups. Hence, lower self-concept score indicates a positive self-concept, so the difference between the real and ideal self-concept in the normal group was less than the other two groups. 

## DISCUSSION

Findings from this study revealed that the groups were significantly different in terms of marital satisfaction. Accordingly, marital satisfaction was higher in the surgery applicant and operated group in comparison to the control group, indicative of the impact of cosmetic surgery on marital satisfaction. In addition, based on marital satisfaction components results, the subjects in the three groups were significantly different in terms of all the marital satisfaction components except for the components of sexual relations, marriage, children, and religious orientation. 

This means that the subjects in the surgery applicant group reported higher satisfaction in most of the components. Accordingly, this applicant group reported higher satisfaction than the operated and control group regarding the personality issues, conflict resolution and leisure activities components, and with higher satisfaction than the control group in terms of the communication component, and a higher satisfaction than the operated group regarding the financial managemen, family and friends components. 

These findings are consistent with the results of the study by Tavassoli *et al.* on the effects of cosmetic surgery on marital satisfaction. The participating women in their study regarded the cosmetic surgery-induced beauty as contributing to greater self-confidence, greater success in married life and higher social status and prestige, as well as a factor to reach power in the family. They showed that, according to Giddens in this sense, human body turns into a place to create and fulfill people’s wishes and aspirations.^19^

Also, the results of this study are in line with the findings of the study by Nikolic *et al.* on motivational factors influencing demand for breast surgery. They showed that the subjects reported a greater sense of femininity, higher attractiveness, less feeling of shame in the presence of men, improved sexual life, and facilitated finding of partners as important motivational factors considered in demand for cosmetic surgery.^20^ In this context, the findings of this study are consistent with the results of Swami *et al.* too.^21^ Accordingly, they examined the relationship between requesting for cosmetic surgery and perfectionism, appearance schemas, satisfaction with romantic styles and relations and suggested that the demand for cosmetic surgery can be predicted based on the dimensions perfectionism, appearance schemas and relation satisfaction.^21^

The findings of this research study are identical to the results emphasizing the importance of psychosocial factors,^5^^,^^22^^,^^23^ and the interaction between psychosocial factors and psychological variables affecting the demand for cosmetic surgery. The results of this study are similar to the McNulty *et al.*’s findings showing that physical attractiveness plays an important role in romantic relations even after initiating a relation.^24^ According to our results, it was shown that planning for cosmetic surgery and, more precisely, the applicants’ expectations for the positive outcome of the cosmetic surgery can have a positive impact on the marital satisfaction.

Our findings showed a statistically significant difference between the operated and surgery applicant and the control groups. The subjects in these two groups received higher self-concept scores. Since the higher self-concept scores reflect the weaker self-concept, the control group had higher levels of self-concept. The results of our study also showed no significant difference in real self-concept scores of the three groups including surgery applicants, operated and control groups. Regarding the ideal self-concept, the results showed that the ideal self-concept was higher in surgery group than the control group and the difference was statistically significant.

According to the results, it was demonstrated that one of the main reasons explaining individuals’ tendency towards cosmetic surgery may be the applicant’s low self-concept. Therefore, the second hypothesis of this study was confirmed. Similarly, it was shown that people who had cosmetic surgery possessed a body image weaker than those who had no interest in cosmetic surgeries.^25^ More specifically, overweight individuals were more interested in liposuction cosmetic surgery and had a body image weaker than the others.^25^

In this regard, the results of this study are in line with the findings of Swami* et al.* They found a significant relationship between gender and age with acceptance of cosmetic surgery.^21^ This relationship can be explained by mediating variables including personality, self-esteem, conformity and physical attractiveness. These results provide a preliminary model integrating personality traits and individual differences in predicting the acceptance of cosmetic surgery.^21^

On the other hand, the results of this study indicated the lack of cosmetic surgery effect on self-concept in women who had cosmetic surgery are not identical with the findings obtained by Soest *et al.*^26^ In their research, they investigated the impacts of plastic surgery on body image, self-esteem and psychological problems prior to and 6 months after performing plastic surgery and found significant improvement in postoperative body image (satisfaction with physical appearance) of the experimental group.^26^ In this regard, it was suggested that individuals reported a better body image after cosmetic surgery procedures. 

According to the results of our study, it can be concluded that the people who had cosmetic surgery as well as being cosmetic surgery applicants in comparison with general population have lower levels of self-concept. These finding have implications for a lot of cosmetic surgery psychiatrists, psychologists, counselors and specialists. Undoubtedly, the identification of psychological factors related to the demand for cosmetic surgery procedures prevents the possible negative consequences of actions induced by psychological factors but not medical necessity.

## CONFLICT OF INTEREST

The authors declare no conflict of interest.
